# Emergence of Next-Generation Sequencing for Laboratory Diagnosis of *Talaromyces marneffei*

**DOI:** 10.1007/s11046-025-01028-3

**Published:** 2025-12-21

**Authors:** Fanfan Xing, Chaowen Deng, Zhendong Luo, Chi-Ching Tsang, Susanna K. P. Lau, Patrick C. Y. Woo

**Affiliations:** 1 Department of Infectious Diseases and Microbiology, The University of Hong Kong–Shenzhen Hospital, Shenzhen, Guangdong China; 2 Department of Radiology, The University of Hong Kong–Shenzhen Hospital, Shenzhen, Guangdong China; 3 School of Medical and Health Sciences, Tung Wah College, Homantin Hong Kong, China; 4 Department of Microbiology, School of Clinical Medicine, Li Ka Shing Faculty of Medicine, The University of Hong Kong, 19/F, Block T, Queen Mary Hospital Compound, Pokfulam, Hong Kong, China; 5 Doctoral Program in Translational Medicine and Department of Life Sciences, National Chung Hsing University, 145 Xingda Road, South District, Taichung, 402 Taiwan; 6 The iEGG and Animal Biotechnology Research Center, National Chung Hsing University, Taichung, 402 Taiwan

**Keywords:** *Talaromyces marneffei*, Next-generation sequencing, Laboratory diagnosis, Rapid diagnosis

## Abstract

The first case of *Talaromyces marneffei* infection diagnosed by next-generation sequencing (NGS) was reported in 2018. By 31st December 2024, the number of *T. marneffei* infections picked up by NGS has increased to a total of at least 241. Most cases were reported from China, reflecting both the regional importance of this infection and its widespread use of NGS for laboratory diagnosis of infectious diseases. Among the cases with clinical details reported, 136 were HIV-negative, which was probably the main reason why *T. marneffei* was not suspected and the diagnosis was subsequently made with the use of NGS. For these 136 HIV-negative patients, 62 were confirmed to have other forms of immunodeficiencies, such as anti-interferon gamma autoantibodies and post-renal/liver transplantation. The clinical presentations were usually non-specific. At least 18 patients were clinically diagnosed as tuberculosis, and empirical anti-tuberculosis therapy was prescribed to 13 patients and NGS was performed because they did not respond to or deteriorated while on anti-tuberculosis treatment. Apart from infectious diseases, at least another 11 patients were initially misdiagnosed to have benign or malignant tumours, the most common being carcinoma of the lung. The median time for detection of *T. marneffei* by NGS was 2 (range 1–6) days, which is significantly shorter than the median time for detection by fungal culture [7 (range 5–17) days] (*P* < 0.0001 by Mann–Whitney U test). NGS has emerged as a promising diagnostic tool for *T. marneffei* infections, especially for picking up cases in HIV-negative patients and rapid diagnosis.

**Supplementary Information:**

The online version contains supplementary material available at 10.1007/s11046-025-01028-3.

## Introduction

*Talaromyces marneffei* is the most significant pathogenic thermally dimorphic fungus responsible for causing systemic mycosis in Southeast Asia. When first isolated from laboratory Chinese bamboo rats (*Rhizomys sinensis*) by Capponi, Segretain and Sureau of Institut Pasteur de Dalat in 1955, this fungus was called *Penicillium marneffei* [1]. It was found to have led to spontaneous disseminated infection affecting the reticuloendothelial system in three of the rats, which became fatal. Segretain’s group described their new discovery as a *Penicillium*-like fungus. Hence it was first named “*P. marneffei*” and was classified in *Penicillium* section *Biverticillium,* following Biourge’s taxonomy, or section *Asymmetrica* subsection *Divaricata,* following Raper’s and Thom’s taxonomy [1]. Based on its phenotypic characteristics, this fungus had been affiliated to the genus *Penicillium*, for over 50 years, with a stable taxonomy until the early 2010s. In 2011, “*P. marneffei*” was renamed “*T. marneffei*”, on the basis of phylogenetic analyses inferred from the RNA polymerase II largest subunit gene (*RPB1*) and internal transcribed spacer (ITS) region as well as extrolite profiling. “*P. marneffei*”, together with other members of *Penicillium* subgenus *Biverticillium*, was transferred to the genus *Talaromyces* [2]. This transfer was further supported by phylogenetic analysis using mitochondrial genomes [3].

While this fungus was first identified in Chinese bamboo rats that are native to the Central Highlands of Vietnam, *T. marneffei* was subsequently cultured from not only other bamboo rat species but also the soil from their burrows, which are regarded as significant enzootic and environmental reservoirs of the fungus, respectively [4–6]. The importance of *T. marneffei* is that at present, it is the only member of the genus found to be able to cause invasive infections in both animals and humans. Infection caused by *T. marneffei* is endemic in tropical regions, particularly Thailand, Vietnam, Northeastern India, Southern China, Hong Kong, Taiwan, Laos, Malaysia, Myanmar, Cambodia and Laos [7]. Research in the past indicated that nearly all *T. marneffei* infections in humans were primarily linked to acquired immunodeficiency syndrome (AIDS) caused by human immunodeficiency virus (HIV) infection [7, 8]. For many decades, this linkage led some regions such as Hong Kong and Southern China to regard *T. marneffei* infection as one of the three main AIDS-defining opportunistic infections, along with tuberculosis and cryptococcosis [9, 10]. However, better understanding of the epidemiology of *T. marneffei* infection has developed in recent years, mainly as a result of improved treatments of HIV infection using highly active antiretroviral therapy and control of the HIV/AIDS epidemic with other measures. In the clinical setting, more and more *T. marneffei* infection cases have been diagnosed and reported in non-HIV-infected patients with other immunocompromising conditions, including primary immunodeficiency syndromes such as anti-interferon gamma autoantibodies, hyper IgE syndrome and hyper IgM syndrome [11–13], or with non-HIV causes of secondary immunodeficiencies such as high dose corticosteroid, organ and bone marrow transplantation and targeted therapies [14–16].

Diagnosis of *T. marneffei* infection is however no easy task because its clinical presentation is extremely non-specific. The diagnosis process therefore often requires clinicians to have the relevant experience coupled with a strong level of suspicion in individual infection cases so that they would order the appropriate laboratory tests to be done and alert the clinical microbiology laboratory on such a suspicion [17, 18]. As a high index of suspicion is involved in diagnosis, it is, more often than not, especially difficult in geographical regions that are not endemic with this fungus. In cases where the appropriate laboratory tests are requested, the traditional approach is to isolate the fungus and demonstrate its characteristic morphological features such that diagnosis of *T. marneffei* infection could be confirmed [17, 19]. However, it typically takes at least five days to isolate *T. marneffei* from clinical specimens even if it could be done successfully. While serological diagnosis is useful and can complement fungal culture, such tests are not available in most laboratories.

The development of next generation sequencing (NGS) has gradually but fundamentally changed the traditional approach to diagnosis of infectious diseases. Continuous technological advancement in NGS has made it powerful enough to sequence all DNA present in a clinical sample, so it could pick up all pathogens and supposedly identify all infectious agents including bacteria, fungi, DNA viruses and parasites [20, 21]. This capability offers unprecedented value to laboratory diagnosis. The need to start the process of diagnosis with a suspicion of a medical condition by the clinician is no longer absolutely necessary as in the traditional patient care approach. Using *T. marneffei* infection as an example, even in cases where a clinician does not suspect it as the cause of the non-specific clinical presentations in a patient and hence is not prepared to order the relevant laboratory test specifically for *T. marneffei* [19], the laboratory can identify all possible pathogens, including *T. marneffei*, using NGS. This new approach enabled by NGS is particularly important in cases where a patient shows pyrexia of unknown origin or other culture-negative infectious disease symptoms. The significance of NGS to both clinicians and laboratories is especially obvious in cases involving infectious agents that are difficult to culture, rarely seen, or even novel pathogens that a clinician could not have suspected just from the patient’s symptoms. The usefulness of NGS in this regard is similar to positron emission tomographic scan in radiology, which could help identify the organ/system in patients with pyrexia of unknown origin. In this article, we reviewed all patients with *T. marneffei* infection reported in the English literature. Their epidemiology and clinical presentations were analyzed and the role of NGS for rapid diagnosis of *T. marneffei* infection was also discussed.

## Methods

A literature search of the PubMed database was performed on 1st January, 2025 using the keywords (“*Penicillium marneffei*” OR “*Talaromyces marneffei*”) AND (“next generation sequencing” OR “NGS” OR “metagenomics” OR “mNGS”), which resulted in 57 articles being retrieved. Of these, 48 were included in this review after manual examination as they represented case reports [22–69], series or cohorts while the remaining publications were excluded since they were considered out of scope or were review articles. If two patients who came from the same hospital were found to have the same age, sex, and clinical presentations, they were regarded as duplicates and one of them was excluded from the analysis.

## Epidemiology and Emerging Role of NGS for Laboratory Diagnosis of *T. marneffei*

**Fig. 1:**
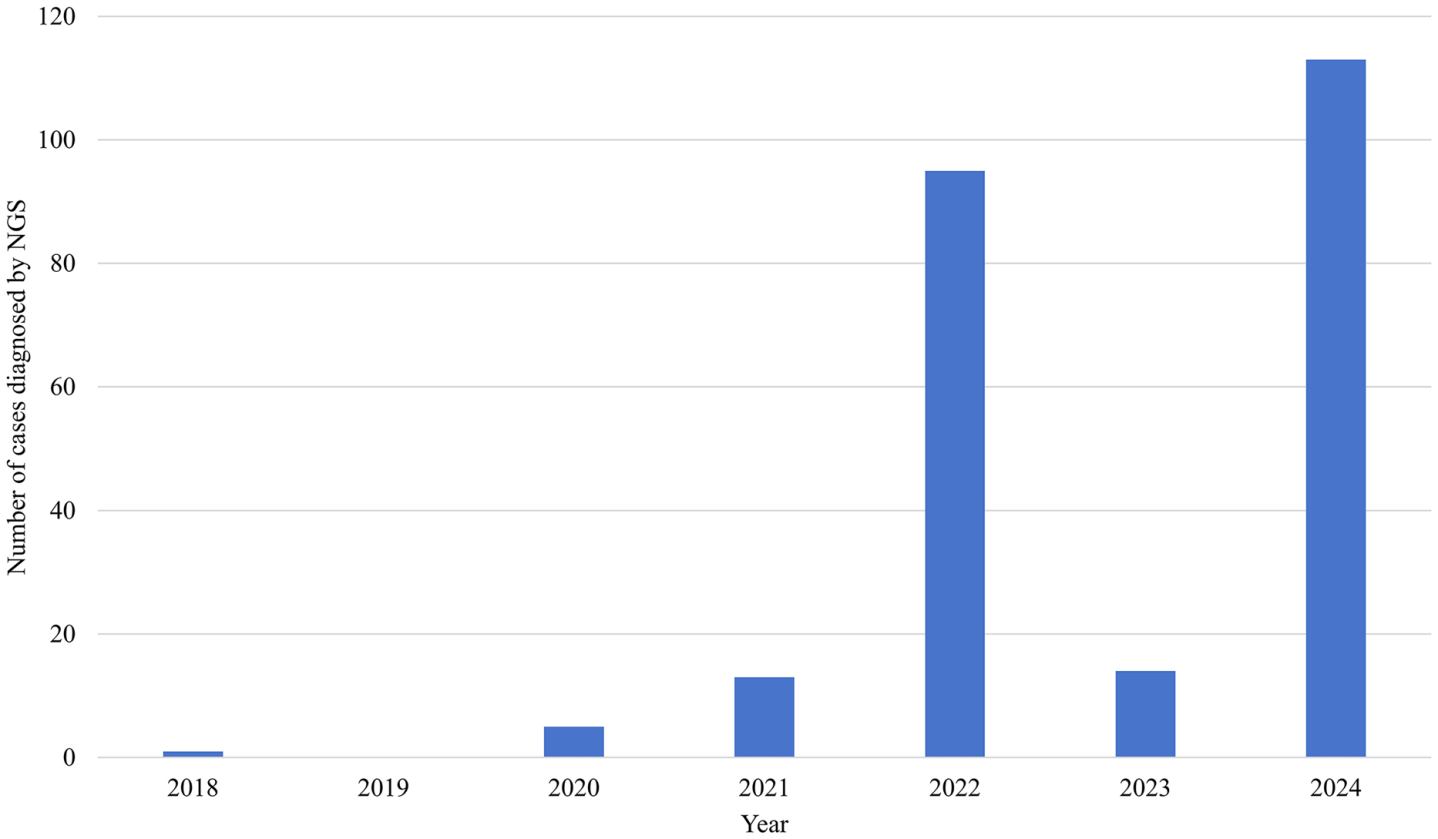
Temporal distribution of *Talaromyces marneffei* infections diagnosed by NGS

The first case of *T. marneffei* infection diagnosed by NGS was reported in 2018 [22]. It described a 22-year-old Chinese man with chronic pneumonia syndrome, chronic osteomyelitis, brain abscess and soft tissue infection. Bronchoalveolar lavage, cerebrospinal fluid, bone marrow, blood and skin lesion sent for NGS analysis were all positive for *T. marneffei* sequence reads. These findings were also further confirmed by nested polymerase chain reaction. The disseminated *T. marneffei* infection responded promptly to intravenous amphotericin B and maintenance oral itraconazole treatment. Since then, the number of *T. marneffei* infections picked up by NGS and reported in the English literature has increased rapidly in the subsequent years (Fig. 1), accumulating to a total of 241 at the time of writing (1st January 2025) (Supplementary Table). Most of the cases were reported from China, reflecting both the regional importance of this infection as well as the widespread use of NGS for laboratory diagnosis of infectious diseases in China as compared to other countries in Southeast Asia, such as Thailand, Vietnam and Cambodia, where *T. marneffei* is prevalent. In fact, after the publication with the title “Expert consensus on the clinical application of metagenomic next-generation sequencing (mNGS) for pathogen detection in infections in China” in 2020 [70], the use of NGS for laboratory diagnosis of infectious diseases has increased rapidly in China, giving to the high number of cases in 2022 and 2024 (Fig. 1). The trough in 2023 was probably due to COVID-19 in China, which had lasted for a few years involving near-complete lockdown, resulting in skewing of resources away from non-COVID-19 matters, including the use of NGS for laboratory diagnosis of infectious diseases in general.

**Fig. 2:**
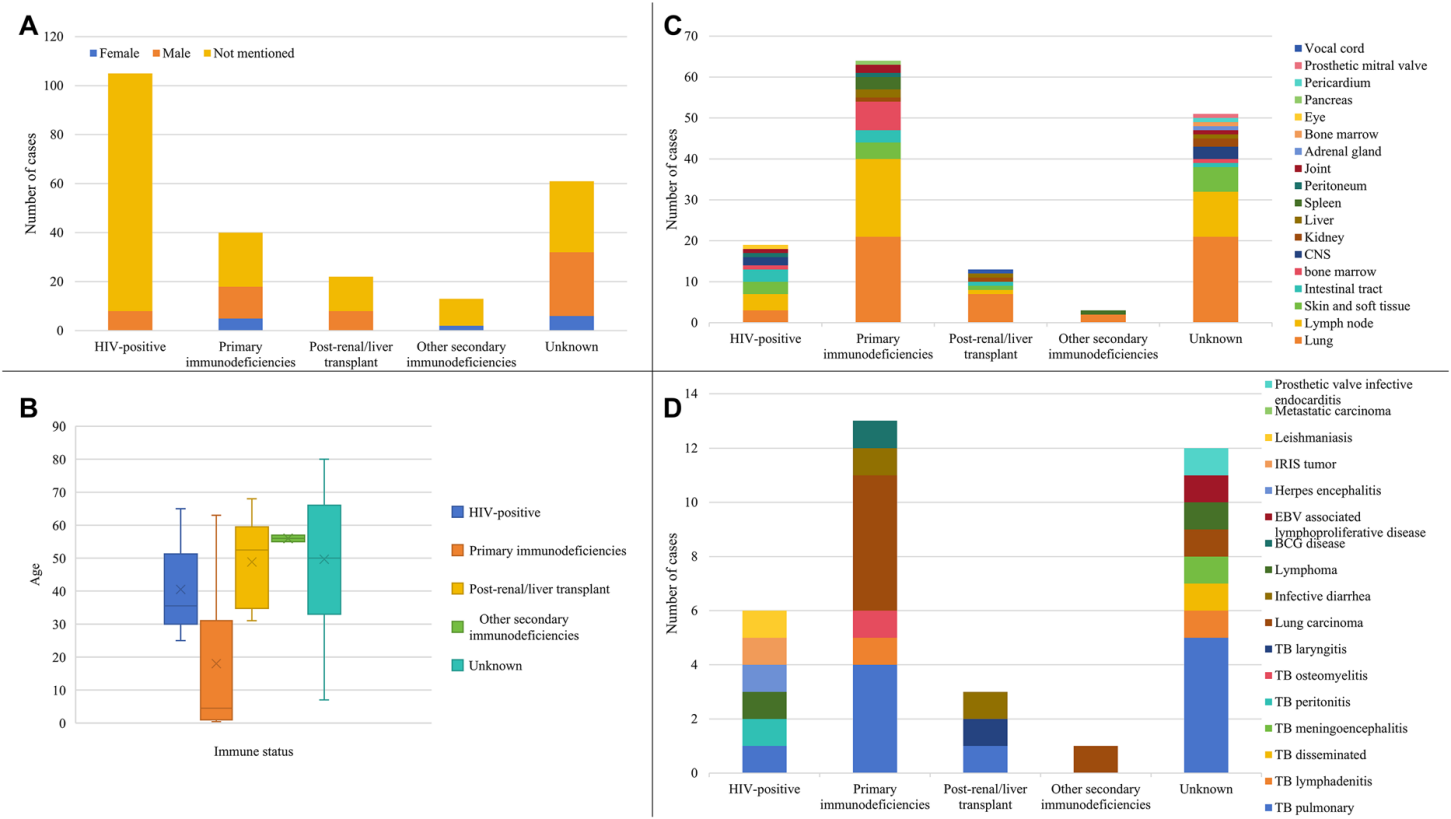
Characteristics of *Talaromyces marneffei* infections diagnosed by NGS in patients with different immune status. Panel A, Sex distribution. Panel B, Age distribution. Middle line in the box represents the median, X in the box represents the mean, edges of the box represent the interquartile range, and short lines at the upper and lower ends represent the range. Panel C, Target organs and/or systems involved by *T. marneffei*. Panel D, Initial diagnosis before performing NGS for cases misdiagnosed as other infections or tumours

Among the cases with demographic details reported, 50 were males and 13 were females (Fig. 2A), giving rise to a rough male to female ratio of 4:1. The median age was 43 (range 0.42–80). One hundred and thirty-six (56.4%) of the 241 patients were HIV-negative (Fig. 2B), which was probably the main reason why *T. marneffei* was not suspected in the first place and hence the diagnosis could only be made with the use of NGS. For these 136 HIV-negative patients, 62 were confirmed to have other forms of immunodeficiencies, including adult onset immunodeficiency syndrome secondary to anti-interferon gamma autoantibodies (n = 23), immunosuppressive treatment after renal or liver transplantation (n = 22), *STAT3* mutation (n = 4), *CD40LG* mutation (n = 4), *CARD9* mutation (n = 3), *STAT1* mutation (n = 3), *IL12RB1* mutation (n = 2), *TSC2* mutation (n = 1), and *IFNGR1* mutation (n = 1).

## Clinical Presentations

Among the 69 patients with *T. marneffei* infections diagnosed by NGS who had their clinical details reported (Fig. 2C), the end organs involved included the lungs in 54 (78.3%) cases, lymph node in 35 (50.7%) cases, skin and soft tissue in 14 (20.3%) cases, intestinal tract in 8 (11.6%) cases, bones in 9 (13.0%) cases, central nervous system in 5 (7.2%) cases, joints in 4 (5.8%) cases, kidneys in 4 (5.8%) cases, liver in 4 (5.8%) cases, spleen in 4 (5.8%) cases, peritoneum in 2 (2.9%) cases, adrenal glands in 1 (1.4%) case, bone marrow in 1 (1.4%) case, eyes in 1 (1.4%) case, pancreas in 1 (1.4%) case, pericardium in 1 case (1.4%), prosthetic mitral valve in 1 (1.4%) case and vocal cords in 1 (1.4%) case.

The clinical presentations of the *T. marneffei* infections were usually non-specific. Since the patients were heavily immunocompromised, they were highly susceptible to a variety of infections, such as tuberculosis. Since both tuberculosis and *T. marneffei* infections present frequently as chronic pneumonia syndrome (three quarters of patients had lung involvement), non-specific systemic infections, or even pyrexia of unknown origins, a case of *T. marneffei* infection could easily be mis-diagnosed clinically as tuberculosis, as the incidence of tuberculosis is much higher than that of talaromycosis in both HIV-positive and HIV-negative populations. In fact, in our review, we found that at least 18 patients were clinically diagnosed as tuberculosis (Fig. 2D). These included pulmonary tuberculosis in cases 5, 7, 13, 18, 19, 20, 96, 101, 117, 120 and 135 [26, 28, 32, 35–37, 41, 42, 53, 56, 65], tuberculosis lymphadenitis in cases 12 and 124 [32, 56], disseminated tuberculosis in case 1 [22], tuberculous meningoencephalitis in case 4 [25], tuberculous peritonitis in case 9 [30], tuberculous osteomyelitis in case 97 [41], and tuberculous laryngitis in case 111 [49]. Furthermore, empirical anti-tuberculosis therapy was prescribed to 13 patients and NGS was performed because they did not respond to or deteriorated while on the treatment for tuberculosis. In addition to tuberculosis (Fig. 2D), two patients were misdiagnosed as infective diarrhea (cases 100 and 116) [41, 52] and one patient each as prosthetic valve infective endocarditis (case 130) [60], Epstein-Barr virus associated lymphoproliferative disease (case 11) [32], herpes encephalitis (case 21) [38], Bacillus Calmette-Guerin disease (case 95) [41], and visceral leishmaniasis (case 108) [47] before NGS analysis results confirmed the diagnosis of talaromycosis. The difficulty in *T. marneffei* diagnosis is clearly illustrated in one of the reports, where a 33-year-old male frequent traveller with on-and-off unprotected sexual behaviour, presented with non-specific symptoms, had suspicious “Leishman-Donovan inclusion bodies” found during microscopic examination of his Wright’s stained bone marrow aspirate smear [47]. He was transferred to a second hospital, where another bone marrow aspirate examination revealed similar findings, and a preliminary diagnosis of Kala-azar was entertained. However, Leishmania antibody test was negative and subsequent NGS analysis of the bone marrow aspirate revealed sequence reads of *T. marneffei*, but none of *Leishmania*. Re-analysis of the bone marrow aspirate smear showed that the suspicious “Leishman-Donovan inclusion bodies” observed were the intracellular and extracellular basophilic yeast form of *T. marneffei*, as shown in other studies and have been used as a means for rapid presumptive diagnosis of this infection [71–73]. Apart from infectious diseases, at least another 11 patients with *T. marneffei* infections eventually diagnosed by NGS were initially mis-diagnosed to have benign or malignant tumours (Fig. 2D), the most common being carcinoma of the lung (cases 118, 122, 124, 125, 126, 128 and 129) [54, 56, 58, 59], as talaromycosis often presented as chronic pneumonia syndrome (cases 2, 7, 8) [23, 28, 29], followed by lymphoma (cases 3 and 10) [24, 31], mediastinal tumour (case 133) [63], and iris tumour of the eye (case 16) [33].

## Rapid Diagnosis of *T. marneffei* Infections by NGS

**Fig. 3:**
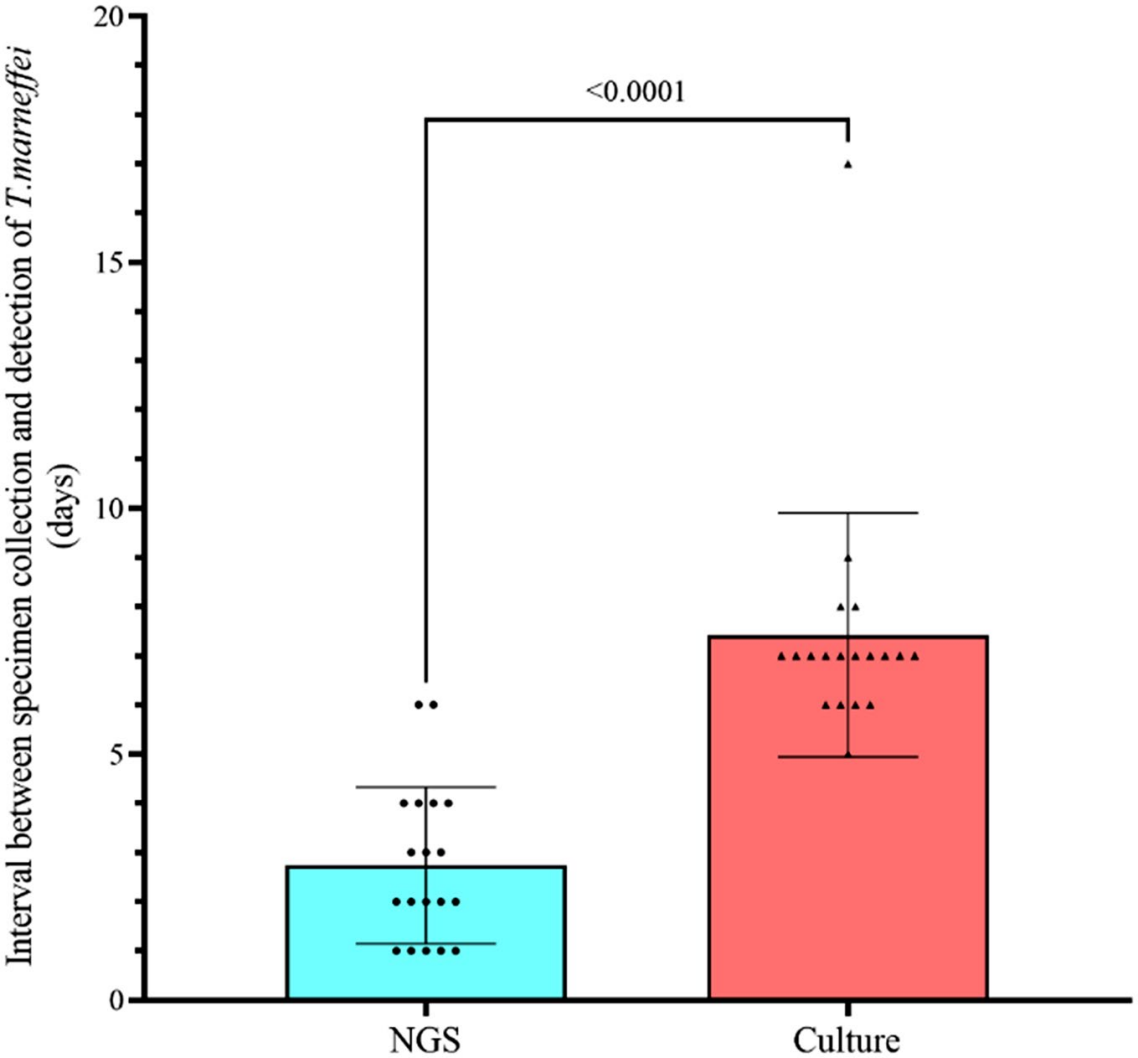
Comparison of time taken for diagnosis of *Talaromyces marneffei* infection by NGS and culture for 19 cases with data available

Rapid diagnosis of infectious disease is crucial in commencing the correct treatment and avoiding the use of unnecessary antimicrobials as well as instituting the necessary infection control measures. In our experience, the turn-around time for NGS analysis of a clinical sample for microorganisms is 1–2 days, which is much shorter than the time required for isolating the difficult-to-grow and slow growing pathogens. In this review, among all the patients with *T. marneffei* infections diagnosed by NGS, details on the time required for generating the NGS results and that for fungal culture were reported in 19 cases. For these 19 cases, the median time for detection of *T. marneffei* by NGS was 2 (range 1–6) days, which is significantly shorter than the median time for detection by fungal culture [7 (range 5–17) days] (*P* < 0.0001 by Mann–Whitney U test) (Fig. 3). Such a small *P* value indicated that in addition to picking up difficult-to-diagnose cases of *T. marneffei* infections, NGS is also of paramount importance for its rapid diagnosis. Furthermore, this is in line with our observation in a recent study on laboratory diagnosis of *T. marneffei i*nfections in renal transplant recipients, which showed that the time taken for diagnosis by mNGS [median 1 (range 1–2) day] was significantly shorter than that for fungal culture [median 6 (range 3–15) days] (*P* = 0.002) [61]. In fact, for the index case described in that study, *T. marneffei* was isolated only because prolonged incubation of the bronchoalveolar lavage sample of the patient was requested after *T. marneffei* sequence reads were detected during NGS analysis of the bronchoalveolar lavage specimen [61]. If NGS analysis was not performed, the bronchoalveolar lavage would have been discarded and the diagnosis of talaromycosis will be delayed.

## Microbes Co-Detected by NGS and Interpretation of Laboratory Results

**Fig. 4:**
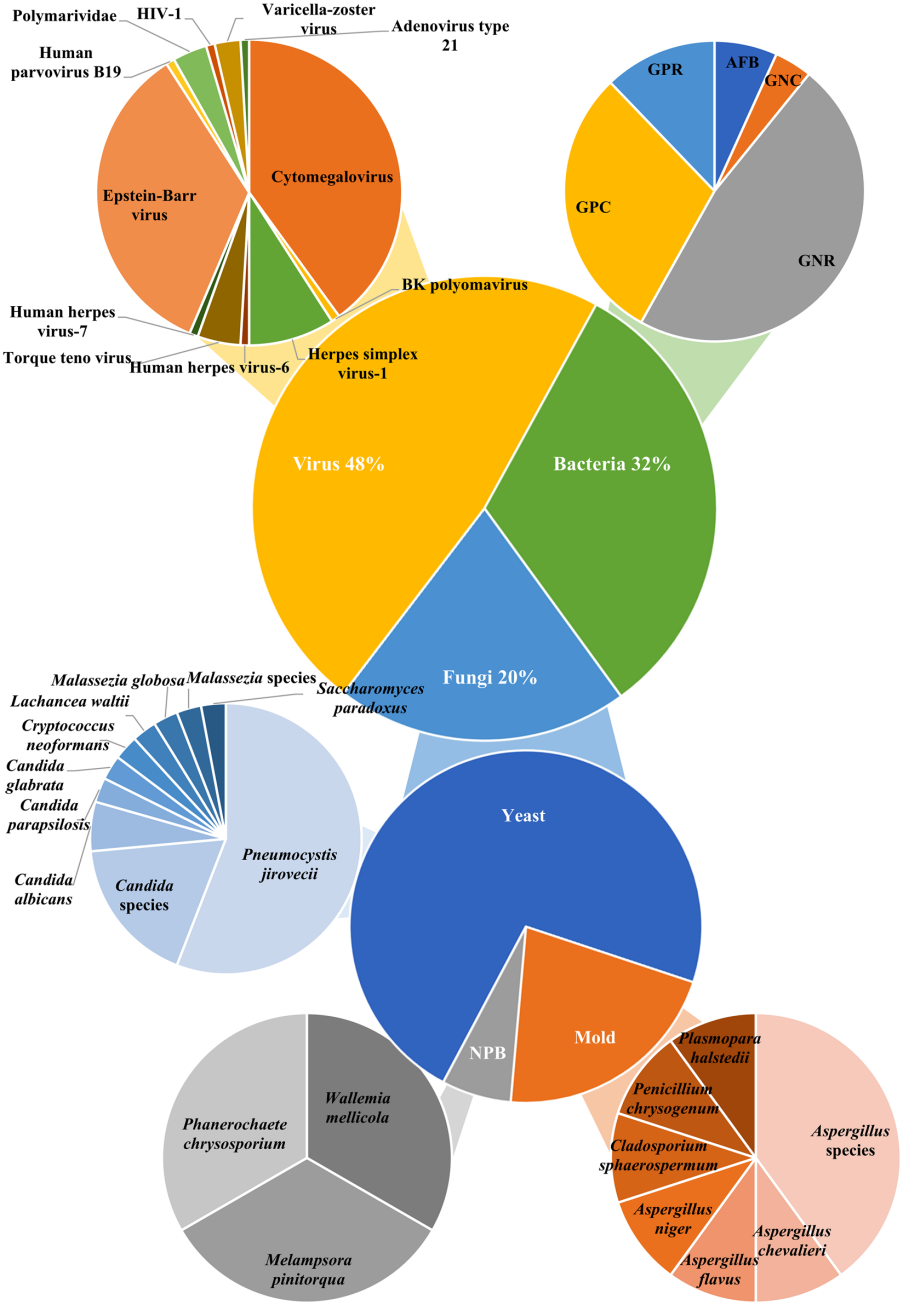
Microorganisms co-detected with *Talaromyces marneffei* in NGS analysis. GPC, gram positive cocci; GPR, gram positive rods; AFB, acid-fast bacilli; GNC, gram negative cocci; GNR, gram negative rods; NPB, non-pathogenic basidiomycetes

Since NGS is such a sensitive technology that it can detect the nucleic acids of any microorganism present in a clinical sample, in addition to the target organism, other microbes are often co-detected [74, 75]. For example, in one of our previous studies that investigated the use of NGS for laboratory diagnosis of culture-negative meningitis and encephalitis, nucleic acids of more than one microorganism were observed in the cerebrospinal fluid of 12 out of the 14 patients, and five of them had six or more microbes found in their specimens [74]. Among the reports reviewed in the present article, co-detection of other microorganisms has been mentioned in 93 [22, 24, 26, 30, 32–39, 41, 42, 45, 50, 51, 53, 56, 61, 64, 66, 68, 69]. In these 93 samples, a median of 2 (range 1–21) microbes were found. Viruses were the most common group of microbes detected, followed by bacteria and fungi (Fig. 4). Among the virus sequences, 88% were from herpesviruses [cytomegalovirus (40%), Epstein-Barr virus (34%), herpes simplex virus-1 (9%), varicella zoster virus (3%), human herpes virus-6 (1%) and human herpes virus-7 (1%)]. The abundance of herpesvirus sequences in NGS analysis was also observed in other NGS studies. For example, in our previous study on the use of NGS for diagnosis of *Pneumocystis jirovecii*, herpesviruses were observed in 36 (64%) of the 56 samples, with cytomegalovirus found in 15 (27%), Epstein-Barr virus in 22 (39%), herpes simplex virus-1 in 5 (9%), human herpes virus-6 in 4 (7%) and human herpes virus-7 in 21 (38%) [76]. For the fungal sequences, 72% were from yeasts. Interestingly, more than half (56%) of the yeast sequences found were from *P. jirovecii*, which was almost twice more common than from the *Candida* species (29%).

Although some of the microbes detected (e.g. *Mycobacterium tuberculosis*, salmonella, and HIV-1) were likely to be co-pathogens, interpretation of the sequences for most of the other additional microbes detected often pose challenges, as they may or may not be co-pathogens in the particular patient [33, 41, 51, 56]. For example, in a 38-year-old HIV-positive male patient who had fever, rash, neurological symptoms and left elbow arthritis, both *T. marneffei* and salmonella sequence reads were detected in the elbow joint fluid of the patient [51]. Since a group D salmonella was also isolated from his blood and cerebrospinal fluid samples, it supported that the salmonella sequence reads detected in the joint fluid probably represented genuine infection. Similar to all other microbiological tests, NGS results must be interpreted along with the specific clinical context discreetly in order to avoid over-treating patients with unnecessary antimicrobials. It is noted that despite 40% of the patients with *T. marneffei* infections diagnosed by NGS were HIV-positive, only one article mentioned that HIV-1 sequences were detected in the NGS analysis results [33]. This is because in China, only the Center of Disease Control or other authorized agencies were permitted to report confirmed results on HIV status, such as HIV antibody and HIV RNA. In this review, it was observed that most of the patient samples were sent to private diagnostic laboratories for NGS analysis, which explained why the HIV-1 sequence reads were removed from the laboratory reports. In the only case from which HIV-1 sequence reads were observed and reported, the HIV-1 sequences were found in the aqueous humor sample of the patient, who was initially mis-diagnosed as iris tumour of the eye (case 16) [33].

## Concluding Remarks

Since its discovery in the 1950s, isolating the fungus in clinical samples and demonstration of its thermal dimorphic features and diffusible red pigment production has been the mainstay of laboratory diagnosis of infections associated with *T. marneffei*. With a surge of clinical cases in the 1980s and 1990s due the HIV pandemic, scientists have been continuously looking for rapid diagnostic tools to supplement the traditional fungal culture. A few years ago, NGS has emerged as a promising diagnostic tool for talaromycosis, especially for picking up cases in HIV-negative patients and rapid diagnosis. With further improvement and cost reduction in the NGS platforms and more streamlined and user-friendly bioinformatics analysis tools, it is anticipated the NGS will play an unprecedentedly crucial role for expedient diagnosis and management of *T. marneffei* infection.

## Supplementary Information

Below is the link to the electronic supplementary material.

Supplementary file1 (DOCX 39 kb)
