# Supplementary material for: Emergence of Next-Generation Sequencing for Laboratory Diagnosis of Talaromyces marneffei
Source: Mycopathologia. 2025 Dec 21;191(1):12. doi: 10.1007/s11046-025-01028-3 (PMC12719348; doi:10.1007/s11046-025-01028-3)
Supplement: Supplementary file 1 — Supplementary file1 (DOCX 39 kb) [file 11046_2025_1028_MOESM1_ESM.docx]

**Supplementary Table. Reported cases of *Talaromyces marneffei* infection diagnosed by next-generation sequencing**

| **Case No.** | **Year** | **Sex** | **Age (year)** | **Underlying/predisposing medical conditions for *T. marneffei* infection** | **Clinical manifestations** | **Clinical syndrome(s) of *T. marneffei* infection** | **NGS (platform if mentioned)** | **Outcome** |
| --- | --- | --- | --- | --- | --- | --- | --- | --- |
| 1[22] | 2018 | Male | 22 | HIV-negative | Fever, cough, lower extremity weakness, jaundice, rash | Disseminated infection | mNGS (BGISEQ-100) | Survived |
| 2[23] | 2020 | Female | 34 | *STAT3*-mutation | Fever, cough, dyspnea, night sweats | Pneumonia | mNGS (Illumina Nextseq 550Dx) | Survived |
| 3[24] | 2020 | Male | 24 | HIV-negative | Fever, diarrhea, fatigue | Disseminated infection | mNGS | Succumbed |
| 4[25] | 2020 | Female | 33 | HIV-negative | Fever, headache, dizziness, blurred vision, vomiting | Disseminated infection | mNGS | Survived |
| 5[26] | 2020 | Male | 52 | HIV-positive | Fever, cough, lymphadenopathy | Disseminated infection | mNGS | Survived |
| 6[27] | 2020 | Male | 23 | HIV-negative | Fever, skin rash, hepatosplenomegaly, lymphadenopathy | Disseminated infection | mNGS | Survived |
| 7[28] | 2021 | Male | 29 | HIV-negative | Fever, dyspnea, chest pain | Pneumonia | mNGS | Survived |
| 8[29] | 2021 | Male | 24 | *TSC2*-mutation | Cough | Pneumonia | mNGS | Survived |
| 9[30] | 2021 | Male | 33 | HIV-positive | Fever, abdominal pain, night sweats, fatigue, diarrhea, weight loss | Disseminated infection | mNGS (BGISEQ-100) | Survived |
| 10[31] | 2021 | Male | 29 | HIV-positive | Abdominal distension, weight loss | Disseminated infection | mNGS | Survived |
| 11[32] | 2021 | Male | 68 | HIV-negative | Fever, cough, lymphadenopathy, weight loss | Disseminated infection | mNGS (BGISEQ-50 / MGISEQ-2000) | Succumbed |
| 12[32] | 2021 | Female | 43 | HIV-negative | Fever, cough, lymphadenopathy, night sweats | Disseminated infection | mNGS (BGISEQ-50 / MGISEQ-2001) | Survived |
| 13[32] | 2021 | Male | 49 | HIV-negative | Fever, cough, thoracalgia, lymphadenopathy, pericardial effusion, pleural effusion | Disseminated infection | mNGS (BGISEQ-50 / MGISEQ-2002) | Survived |
| 14[32] | 2021 | Female | 45 | HIV-negative | Cough, right sacroiliac articulation pain, night sweats, hemoptysis, skin rash | Disseminated infection | mNGS (BGISEQ-50 / MGISEQ-2003) | Survived |
| 15[32] | 2021 | Male | 54 | HIV-negative | Fever, cough, skin rash, lymphadenopathy, night sweats | Pustulosis and lymphadenopathy | mNGS (BGISEQ-50 / MGISEQ-2004) | Survived |
| 16[33] | 2021 | Male | 25 | HIV-positive | Blurred vision, corneal edema, posterior synechia | Disseminated infection | mNGS (Ion Proton) | Survived |
| 17[34] | 2021 | Male | 7 months | *CARD9*-mutation | Fever, cough, skin rash, lymphadenopathy, lower limb swelling, hepatosplenomegaly | Disseminated infection | mNGS | Survived |
| 18[35] | 2021 | Male | 79 | HIV-negative | Fever, cough | Pneumonia and lymphadenopathy | mNGS | Survived |
| 19[36] | 2021 | Male | 34 | Renal transplant recipient on corticosteroids and immunosuppressants | Cough, hemoptysis, weakness, poor appetite, weight loss | Pneumonia | mNGS | Survived |
| 20[37] | 2022 | Male | 50 | HIV-negative | Fever, cough, night sweats | Disseminated infection | mNGS | Survived |
| 21[38] | 2022 | Male | 65 | HIV-positive | Fever, abnormal behavior, dysphagia, cough | Disseminated infection | mNGS | Survived |
| 22[39] | 2022 | Male | 70 | HIV-negative | Not mentioned | Pneumonia | mNGS | Survived |
| 23[39] | 2022 | Female | 33 | HIV-negative | Not mentioned | Pneumonia | mNGS | Survived |
| 24[39] | 2022 | Male | 46 | HIV-negative | Not mentioned | Pneumonia | mNGS | Survived |
| 25[39] | 2022 | Male | 55 | HIV-negative | Not mentioned | Pneumonia | mNGS | Survived |
| 26[39] | 2022 | Male | 58 | HIV-negative | Not mentioned | Not mentioned | mNGS | Survived |
| 27[39] | 2022 | Male | 52 | HIV-negative | Not mentioned | Not mentioned | mNGS | Survived |
| 28[39] | 2022 | Female | 7 | HIV-negative | Not mentioned | Not mentioned | mNGS | Survived |
| 29[39] | 2022 | Male | 48 | HIV-negative | Not mentioned | Not mentioned | mNGS | Succumbed |
| 30[39] | 2022 | Male | 66 | HIV-negative | Not mentioned | Pneumonia | mNGS | Survived |
| 31[39] | 2022 | Not mentioned | Not mentioned | HIV-negative | Not mentioned | Not mentioned | mNGS | Not mentioned |
| 32[39] | 2022 | Not mentioned | Not mentioned | HIV-negative | Not mentioned | Not mentioned | mNGS | Not mentioned |
| 33[39] | 2022 | Not mentioned | Not mentioned | HIV-negative | Not mentioned | Not mentioned | mNGS | Not mentioned |
| 34[39] | 2022 | Not mentioned | Not mentioned | HIV-negative | Not mentioned | Not mentioned | mNGS | Not mentioned |
| 35[39] | 2022 | Not mentioned | Not mentioned | HIV-negative | Not mentioned | Not mentioned | mNGS | Not mentioned |
| 36-94[40] | 2022 | Not mentioned | Not mentioned | HIV-positive | Not mentioned | Not mentioned | mNGS (MGISEQ-2000 / MGISEQ-50) | Not mentioned |
| 95[41] | 2022 | Female | 3 | *IL12RB1*-mutation | Fever, lymphadenopathy, anemia, hepatosplenomegaly | Disseminated infection | mNGS (Illumina NextSeq 550Dx) | Survived |
| 96[41] | 2022 | Male | 5 months | *IL12RB1*-mutation | Lymphadenopathy | Disseminated infection | mNGS (Illumina NextSeq 550Dx) | Survived |
| 97[41] | 2022 | Male | 2 | *IFNGR1*-mutation | Fever, lower extremity pain | Disseminated infection | mNGS (Illumina NextSeq 550Dx) | Survived |
| 98[41] | 2022 | Female | 11 months | *STAT3*-mutation | Fever, cough, shortness of breath, laryngeal stridor | Pneumonia | mNGS (Illumina NextSeq 550Dx) | Survived |
| 99[41] | 2022 | Male | 2 | *STAT1*-mutation | Fever, nausea, lethargy, dyspnea, hepatosplenomegaly, lymphadenopathy, anemia, thrombocytopenia | Disseminated infection | mNGS (Illumina NextSeq 550Dx) | Survived |
| 100[41] | 2022 | Male | 2 | *CD40LG*-mutation | Fever, diarrhea | Disseminated infection | mNGS (Illumina NextSeq 550Dx) | Succumbed |
| 101[42] | 2022 | Male | 6 | *CD40LG*-mutation and *CARD9*-mutation | Fever, lymphadenopathy | Disseminated infection | mNGS | Survived |
| 102[43] | 2022 | Male | 8 months | *CD40LG*-mutation | Fever, cough | Disseminated infection | mNGS | Survived |
| 103[44] | 2022 | Male | 61 | Renal transplant recipient on corticosteroids and immunosuppressants | Fever, cough, shortness of breath | Pneumonia | mNGS | Succumbed |
| 104[44] | 2022 | Male | 55 | Renal transplant recipient on corticosteroids and immunosuppressants | Fever, shortness of breath | Pneumonia | mNGS | Succumbed |
| 105[45] | 2022 | Male | 80 | HIV-negative | Fever, cough, lymphadenopathy | Disseminated infection | mNGS (Illumina) | Survived |
| 106[45] | 2022 | Female | 57 | Multiple myeloma, hematopoietic stem cell transplantation recipient on corticosteroids | Fever, dizziness, diarrhea, abdominal pain | Disseminated infection | mNGS (Illumina) | Survived |
| 107[46] | 2022 | Male | 51 | Renal transplant recipient on corticosteroids and immunosuppressants | Fever, cough, night sweats | Pneumonia | mNGS | Survived |
| 108[47] | 2022 | Male | 33 | HIV-positive | Fever, cough, fatigue, night sweats, abdominal distension, diarrhea | Disseminated infection | mNGS (Nanopore MinION) | Survived |
| 109[48] | 2022 | Male | 21 | *STAT3*-mutation | Fever, cough, skin rash, lymphadenopathy | Disseminated infection | mNGS | Survived |
| 110[48] | 2022 | Male | 15 | *STAT3*-mutation | Fever, cough, skin rash | Disseminated infection | mNGS | Survived |
| 111[49] | 2022 | Male | 54 | Renal transplant recipient on immunosuppressants | Sore throat, odynophagia | Pneumonia and laryngitis | mNGS | Survived |
| 112-114[50] | 2022 | Not mentioned | Not mentioned | Not mentioned | Not mentioned | Not mentioned | mNGS | Not mentioned |
| 115[51] | 2023 | Male | 38 | HIV-positive | Fever, skin rash, abnormal behavior, neck rigidity, lymphadenopathy, joint swelling and pain | Disseminated infection | mNGS | Survived |
| 116[52] | 2023 | Male | 31 | Renal transplant recipient on corticosteroids and immunosuppressants | Fever, low back pain, diarrhea, melena, lymphadenopathy, hepatosplenomegaly | Disseminated infection | mNGS (Illumina NextSeq 550Dx) | Survived |
| 117[53] | 2023 | Female | 30 | Adult-onset immunodeficiency syndrome secondary to anti-interferon gamma autoantibodies | Fever, cough, chest pain, back pain | Disseminated infection | mNGS (Illumina NextSeq 550) | Survived |
| 118[54] | 2023 | Male | 56 | Adult-onset immunodeficiency syndrome secondary to anti-interferon gamma autoantibodies | Fever, cough, fatigue, night sweats, chest pain | Pneumonia and lymphadenopathy | mNGS | Survived |
| 119[55] | 2023 | Male | 1 | *STAT1*-mutation | Fever, shortness of breath, trachyphonia | Pneumonia, laryngitis and lymphadenopathy | mNGS | Survived |
| 120[56] | 2023 | Not mentioned | Not mentioned | Adult-onset immunodeficiency syndrome secondary to anti-interferon gamma autoantibodies | Fever, cough, shortness of breath, anemia, lymphadenopathy, weight loss | Disseminated infection | mNGS | Survived |
| 121[56] | 2023 | Not mentioned | Not mentioned | Adult-onset immunodeficiency syndrome secondary to anti-interferon gamma autoantibodies | Skin rash, joint pain, headache | Disseminated infection | mNGS | Succumbed |
| 122[56] | 2023 | Not mentioned | Not mentioned | Adult-onset immunodeficiency syndrome secondary to anti-interferon gamma autoantibodies | Cough, fatigue, anorexia, weight loss, anemia | Disseminated infection | mNGS | Survived |
| 123[56] | 2023 | Not mentioned | Not mentioned | Adult-onset immunodeficiency syndrome secondary to anti-interferon gamma autoantibodies | Cough, chest pain, lymphadenopathy, anorexia, weight loss | Disseminated infection | mNGS | Succumbed |
| 124[56] | 2023 | Not mentioned | Not mentioned | Adult-onset immunodeficiency syndrome secondary to anti-interferon gamma autoantibodies | Fever, cough, wheezing, lymphadenopathy | Disseminated infection | mNGS | Survived |
| 125[56] | 2023 | Not mentioned | Not mentioned | Adult-onset immunodeficiency syndrome secondary to anti-interferon gamma autoantibodies | Cough, shortness of breath, chest pain, weight loss | Disseminated infection | mNGS | Survived |
| 126[56] | 2023 | Not mentioned | Not mentioned | Adult-onset immunodeficiency syndrome secondary to anti-interferon gamma autoantibodies | Cough, shortness of breath, chest pain, weight loss | Disseminated infection | mNGS | Survived |
| 127[57] | 2023 | Male | 49 | HIV-positive | Abdominal distension, poor appetite, skin rash | Gastrointestinal ulcer | mNGS | Survived |
| 128[58] | 2023 | Female | 55 | Myasthenia gravis on corticosteroids and immunosuppressants | bone and joint pain | Pneumonia | mNGS | Survived |
| 129[59] | 2024 | Male | 70 | Low-grade small B-cell non-Hodgkin lymphoma | Fever, cough, fatigue, lymphadenopathy | Disseminated infection | NGS | Survived |
| 130[60] | 2024 | Female | 80 | Mitral valve replacement | Fever, shortness of breath, fatigue, systolic heart murmur | Disseminated infection | mNGS | Succumbed |
| 131[61] | 2024 | Male | 37 | Renal transplant recipient on corticosteroids and immunosuppressants | Fever, cough, shortness of breath, subcutaneous nodules | Disseminated infection | mNGS | Survived |
| 132[62] | 2024 | Male | 68 | Renal transplant recipient on corticosteroids and immunosuppressants | Urinary irritation | Disseminated infection | mNGS | Succumbed |
| 133[63] | 2024 | Female | 62 | Adult-onset immunodeficiency syndrome secondary to anti-interferon gamma autoantibodies | Fever, headache, neck pain | Disseminated infection | mNGS | Survived |
| 134[64] | 2024 | Male | 52 | Liver cirrhosis | None | Pneumonia | mNGS | Survived |
| 135[65] | 2024 | Male | 43 | HIV-negative | Fever, cough, joint pain, subcutaneous modules, lymphadenopathy | Disseminated infection | mNGS | Survived |
| 136[66] | 2024 | Male | 63 | *CARD9*-mutation | Abdominal pain, peritoneal irritation | Disseminated infection | mNGS | Succumbed |
| 137[67] | 2024 | Male | 63 | HIV-negative | Fever, cough | Pneumonia and type Ⅰ respiratory failure | mNGS | Survived |
| 138-164[68] | 2024 | Not mentioned | Not mentioned | Adult-onset immunodeficiency syndrome secondary to anti-interferon gamma autoantibodies in 13 cases, *CD40LG*-mutation in 1 case, *STAT1*-mutation in 1 case | Not mentioned | Disseminated infection in 20 cases, pneumonia in 5 cases, skin and soft tissue infection in 1 case | mNGS (Illumina NextSeq) | Survived in 25 cases |
| 165-241[69] | 2024 | Female/Male=19/58 | Not mentioned | HIV positive in 38 cases, liver or renal transplant on corticosteroids and immunosuppressants in 14 cases, hematologic malignancy on immunosuppressants in 6 cases, hematologic malignancy on immunosuppressants in 5 cases | Fever, respiratory symptom, digestive tract symptom, skin lesion, lymphadenopathy | Not mentioned | mNGS (Illumina NextSeq CN500) | Survived in 69 cases, succumbed in 8 cases |
